# Magnesium chelatase subunit D is not only required for chlorophyll biosynthesis and photosynthesis, but also affecting starch accumulation in *Manihot esculenta* Crantz

**DOI:** 10.1186/s12870-023-04224-9

**Published:** 2023-05-16

**Authors:** Xingai Yang, Jie Cai, Jingjing Xue, Xiuqin Luo, Wenli Zhu, Xinhui Xiao, Maofu Xue, Feifei An, Kaimian Li, Songbi Chen

**Affiliations:** Key Laboratory of Ministry of Agriculture for Germplasm Resources Conservation and Utilization of Cassava, Tropical Crops Genetic Resources Institute, Chinese Academy of Tropical Agricultural Science, Haikou, 571101 China

**Keywords:** Cassava (*Manihot esculenta* Crantz), *MeChlD*, VIGS, Chlorophyll synthesis, Photosynthesis, Starch accumulation

## Abstract

**Background:**

Magnesium chelatase plays an important role in photosynthesis, but only a few subunits have been functionally characterized in cassava.

**Results:**

Herein, *MeChlD* was successfully cloned and characterized. *MeChlD* encodes a magnesium chelatase subunit D, which has ATPase and vWA conservative domains. *MeChlD* was highly expressed in the leaves. Subcellular localization suggested that MeChlD:GFP was a chloroplast-localized protein. Furthermore, the yeast two-hybrid system and BiFC analysis indicated that MeChlD interacts with MeChlM and MePrxQ, respectively. VIGS-induce silencing of *MeChlD* resulted in significantly decreased chlorophyll content and reduction the expression of photosynthesis-related nuclear genes. Furthermore, the storage root numbers, fresh weight and the total starch content in cassava storage roots of VIGS-*MeChlD* plants was significantly reduced.

**Conclusion:**

Taken together, MeChlD located at the chloroplast is not only required for chlorophyll biosynthesis and photosynthesis, but also affecting the starch accumulation in cassava. This study expands our understanding of the biological functions of ChlD proteins.

**Supplementary Information:**

The online version contains supplementary material available at 10.1186/s12870-023-04224-9.

## Background

Cassava (*Manihot esculenta* Crantz) is an important food staple for raising nearly a billion people in the tropics, playing vital functions in food security [1]. Cassava not only used as industrial raw materials, but also act as edible food in China, attributed to its starchy roots [2, 3]. In recent decades, China became a major country of cassava production in the world during the long-term cultivation, the harvest area and yield were increased in China [4]. However, the production of cassava in China could not afford the domestic consumption, self-sufficiency rate was only 18%, most of cassava dry slices and starch dependent on import [5]. Therefore, it is an urgence to solve the disadvantaged situation by cultivating cassava varieties harboring the excellent characteristic with high-yield and high starch accumulation. Furthermore, cassava is a typical source-to-sink tuberous crop [6, 7]. Thus, to better understanding the molecular regulation mechanism of chlorophyll biosynthesis is the initial step for cassava breeding.

Chlorophyll is synthesized from Mg-protoporphyrin IX in the biosynthesis pathway. Magnesium chelatase is the first committed enzyme for Mg-porphyrin branch, which catalyzed Mg^2+^ in to protoporphyrin IX by consumption of ATP. Magnesium chelatase consists of at least three different subunits, ChlI, ChlH and ChlD subunits [8]. These subunits have been reported in plants, especially in *Oryza sativa* [9–11], *Nicotiana benthamiana* [12, 13], *Arabidopsis thaliana* [14, 15], *Glycine max* [16–18], *Pisum sativum* [19], *Zea mays* [20], which have specific functions in chlorophyll biosynthesis and photosynthesis. AtChlH protein involves in plastid-to-nucleus signal transduction [21, 22]. Mutations in ChlD also impairs retrograde signaling [23]. Additionally, ChlH and ChlI except ChlD also affect ABA signaling in stomatal guard cell in *A. thaliana* and *N. benthamiana* [24, 25]. These results indicated these subunits have multiple roles in plants. Thus, although the regulatory mechanism of chlorophyll biosynthesis is currently being elucidated in many species, there are still many details that required further investigation in cassava.

Virus-induced gene silencing (VIGS) system was widely and successfully used to characterized gene functions in many plants [26–30]. VIGS is mostly drive on RNA viruses, such as *tobacco rattle virus* (TRV), *tobacco mosaic virus* (TMV), *cucumber mosaic virus* (CMV), *potato virus X* (PVX) and *cassava common mosaic virus* (CsCMV). Especially, TRV and CsCMV are widely used in cassava for rapid gene function analysis [29–32], due to high silencing efficiency, long silencing duration, and obvious virus-induced disease symptoms. A *magnesium chelatase subunit I* (*ChlI*) was successfully silenced in tobacco and different cassava varieties by CsCMV-mediate VIGS system [30]. Therefore, CsCMV mediated-VIGS system can be used as a new tool for rapidly characterized gene functions in cassava.

In this study, *MeChlD* was successfully cloned and characterized, which encodes a magnesium chelatase subunit D. The tissue specific expression, subcellular localization, protein interactions and VIGS-inducing the phenotypes were characterized. These results suggested that MeChlD was chlorophyll-localized is required for chlorophyll biosynthesis, photosynthesis and starch metabolism in cassava.

## Methods

### Plant materials and growth conditions

Cassava variety South China 9 (SC9) was used in this study, which provided from National Cassava Germplasm Repository (Danzhou city, Hainan province). Tobacco (*N. benthamiana*) was provided by Prof. Yao from Institute of Tropical Bioscience and Biotechnology, Chinese academy of Tropical Agricultural Science, which was used in this study to analyzed subcellular localization and protein interactions. All plants were kept in an air-conditioned growth room (22 °C; 16-h photoperiod, 2000 lx light intensity).

### Gene cloning and plasmid construction

Gene sequence of *MeChlD* was cloned using cDNA as a template. DNA sequences encoding proteins (MeChlD, MeChlM and MePrxQ) without signal peptides were cloned into prokaryotic expression vector pET28a (Novagen, Merck).

To analyze localization of *GFP*-tagged MeChlD, the coding sequence of *MeChlD* was PCR-amplified, and then inserted at the *Nco*I site of the binary vector pCAMBIA1302 (yielding a 35S*pro*-*MeChlD*:*GFP* construct).

To examine the interaction relationship between MeChlD and MeChlM/MePrxQ by using the yeast two-hybrid system, the vectors pGBKT7-*MeChlD,* pADT7-*MeChlM*, pADT7-*MePrxQ,* pGBKT7-*MeChlM,* pGBKT7-*MePrxQ and* pADT7-*MeChlD* were constructed and transformed into yeast competent cell AH109, respectively.

To further confirm the interaction by using BiFC, these vectors pNC-BiFC-Enn-*MeChlD* with *N*-terminal enhanced yellow fluorescence protein (nYFP), pNC-BiFC-Ecc-*MeChlM* and pNC-BiFC-Ecc-*MePrxQ* with C-terminal enhanced YFP (cYFP) were constructed by seamless cloning (ClonExpress II One Step Cloning Kit, Vazyme Biotech Co., Ltd), and transformed into *Agrobacterium tumefaciens* GV3101-pSoup-p19.

To investigate the role of *MeChlD*, 300 bp DNA fragment was PCR-amplified using cDNA of SC9 and finally cloned into the a VIGS vector pCsCMV-NC as described [30], yielding pCsCMV-*MeChlD*.

All constructs were verified by DNA sequencing. Primer sequences for plasmid construction are shown in Table S1.

### Identification and bioinformatic analysis of MeChlD

The MW (molecular weight), p*I* (theoretical isoelectric point) and positive/negative charge residues of MeChlD were predicted by ExPASy [33]. The ProtScale program (http://web.expasy.org/ protscale/) was used to analyze the hydrophilicity of the ChlD protein. The gene expression of *MeChlD* was characterized in cassava expression atlas as described [34].

For the identification of MeChlD and related proteins, the amino acid sequence of MeChlD was used as a query sequence for blast at the NCBI (https://blast.ncbi.nlm.nih.gov/Blast.cgi). MEGA X [35] was used to multiple alignment analysis of MeChlD and homologous proteins and a phylogenetic tree was constructed using the Neighbor-joining method.

### Measurement of the net photosynthetic rate in cassava accessions

The net photosynthetic rate under nesting scenarios along with the temperature, CO_2_ concentration, and the photon flux density was measured by LI-6800 portable photosynthesis system (Li-COR company, Nebraska, USA). The required temperature was set 25 °C by temperature controlling module, the CO_2_ injection volume ratio was set 400 μL/L, the photo flux density was set to 1000 μmol/(m^2^·s) by LED light source module, the relative humidity was set 50–70% by water control module. Fully expanded functional leaves of cassava were randomly selected. The timing for measuring was selected at 09:00 and 12:00 am to avoid the effects of stomatal closure on this study.

### The growth of the recombinant proteins under different concentrations of Mg^2+^ treatments

The vectors pET-28a-*MeChlD*, pET-28a-*MeChlM*, pET-28a-*MePrxQ* and pET-28a were transformed into *E. coil* (DE3). These recombinant proteins MeChlD, MeChlM and MePrxQ were treated by different concentrations of magnesium ion as described [36].

### Subcellular localization of MeChlD in tobacco leaves

The vectors pCAMBIA1302-35S*pro*-*MeChlD*:*GFP* and pCAMBIA1302 were transformed into *A. tumefaciens* strain GV3101, and then infiltrated into *N. benthamiana* leaves as described. After 3 days post inoculation (dpi), GFP signals (395–509 nm) were detected using a laser confocal microscope.

### Y2H assay

These vectors pGBKT7-*MeChlD*/pADT7, pGBKT7-*MeChlM*/pADT7 and pGBKT7-*MePrxQ*/pADT7, pGBKT7-*MeChlD*/pADT7-*MeChlM*, pGBKT7-*MeChlD*/pADT7-*MePrxQ* and pGBKT7-*MeChlM*/pADT7-*MePrxQ* were transformed into yeast competent cell AH109, and then the positive clones were screened on the selective SD dropout medium (SD-Leu-Trp and SD-Leu-Trp-His-Ade).

### Bimolecular fluorescence complementation (BiFC) analysis

These vectors pNC-BiFC-Enn-*MeChlD*, pNC-BiFC-Ecc-*MeChlM* and pNC-BiFC-Ecc-*MePrxQ* were transformed into *A. tumefaciens* strain GV3101-psoup-p19, and then infiltrated into *N. benthamiana* leaves (growth of 30 days) as described [37]. The yellow fluorescence (514–527 nm) in the infiltrated leaf areas was examined using a laser confocal microscope.

### VIGS assay in cassava

The best VIGS target region of *MeChlD* CDS sequence (Manes. 14G005700) was analyzed by online software (SGN VIGS Tool), and then we amplified this region (274–573 bp of CDS sequence) and constructed the vectors pCsCMV-*MeChlD*. The vectors pCsCMV-*MeChlD* and pCsCMV-NC were transformed into *A. tumefaciens* strain GV3101-pSoup-p19, and then infiltrated the leaves of cassava variety SC9 by a syringe injection as described [30].

The VIGS-mediated silent plants were grown in the air-condition room (22 °C; 16-h photoperiod). After 3 weeks post inoculation, the expression level of *MeChlD* in silent plants of cassava was measured by qRT-PCR to further calculate the silence efficient. Currently, chlorophyll content in different silent plants of cassava was performed as described [38]. The content of the total starch was measured as described [32].

### RNA extraction and qRT-PCR verification

Total RNA was extracted from cassava leaves using a RNAprep Pure Plant plus Kit (Tiangen, China). One-Step gDNA Removal and cDNA Synthesis SuperMix (TransGen, China) was used for first-strand cDNA synthesis. The reactions solutions were performed in 10 mL volume in Real-time Thermal Cycler fluorescence quantification apparatus (Thermo Fisher Scientific Inc., Göteborg, Sweden). *MeActin* was used as an internal reference gene. All samples were performed in triplicate per sample. The relative expression levels of genes were calculated by the formula 2^−ΔΔCT^ method. The qRT-PCR primers were shown in Table S1.

### Statistical analysis

Microsoft Excel 2010 was used for data processing; SPSS 23.0, DPS 7.0 and Kruskal–Wallis software was used for data analysis; GraphPad 6.0 and Origin2021 software were used to create experimental figures.

## Results

### MeChlD encodes a magnesium chelatase subunit D

A magnesium chelatase subunit D, *MeChlD*, was successfully amplified and sequencing in cassava variety SC9 (Figure S1). The length of coding sequence is 2250 bp, encodes 749 amino acids. The information of MV, p*I* and positive/negative charge residues were 82.27 kDa, 5.81, 100 and 92, respectively (Table S2). Bioinformatic analysis indicated that MeChlD belongs to stable protein and hydrophilic protein (Figure S2).

**Fig. 1:**
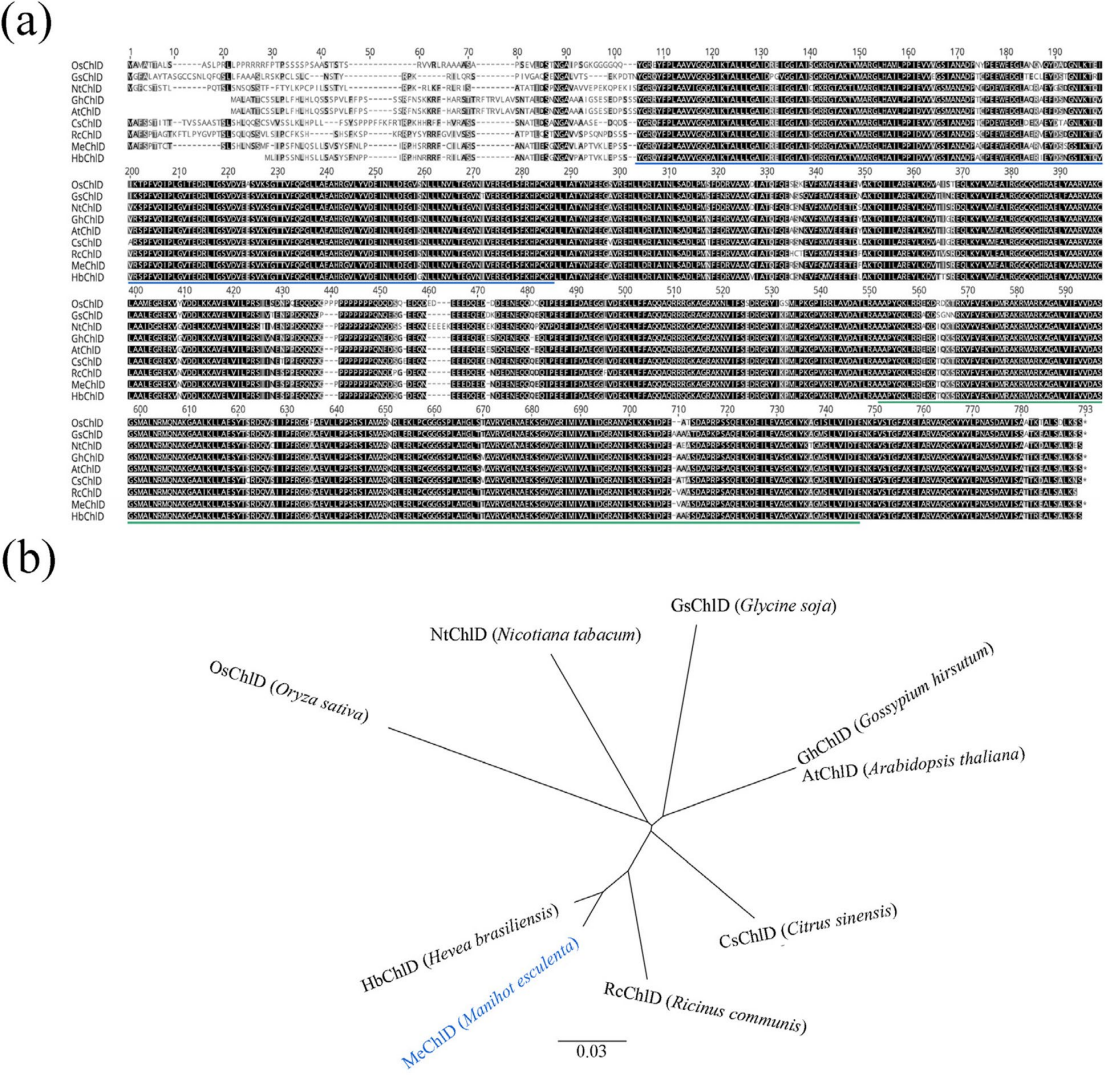
Alignment and phylogenetic analysis of MeChlD and MeChlD-related proteins in plants. **a** Alignment of amino acid sequences of MeChlD and MeChlD-related proteins. The alignment was performed with GENEIOUS software (https://www.geneious.com/). Identical amino acid residues are indicated with a black background, homologous residues with a grey background and dashes indicate gaps. The blue line and green line indicate ATPase domain and vWA structural domain, respectively. **b** A corresponding phylogenetic tree of MeChlD and related amino acid sequences. MeChlD characterized in this study are highlighted in blue. The tree was constructed by using the Neighbor-joining method. The horizontal bar represents a distance of 0.03 substitutions per site

To further characterize ChlD protein, we performed a study with ChlD of *M. esculenta* and homologues from HbChlD (*Hevea brasiliensis*), RcChlD (*Ricinus communis*), AtChlD (*A*. *thaliana*), NtChlD (*N*. *tabacum*), OsChlD (*Oryza sativa*), GsChlD (*G*. *soja*), CsChlD (*Citrus sinensis*) and GhChlD (*Gossypium hirsutum*). An amino acid sequence alignment of these proteins and a corresponding phylogenetic tree is shown (Fig. 1). Most of these ChlD proteins have conservative domains, ATPase domain without ATPase activity, and vWA structural domain with binding site for metal ion (Fig. 1a). MeChlD shows more than 81% identities with these proteins, especially has the closer relationship with HbChlD (96.74% identities) and RcChlD (90.60% identities) (Fig. 1b).

### Mg^2+^ affects the growth of the recombinant protein MeChlD

**Fig. 2:**
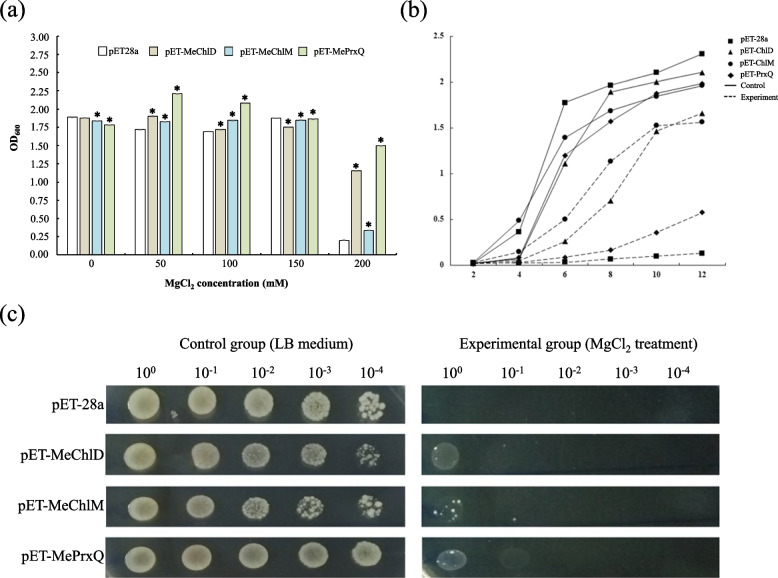
The growth of the recombinant proteins MeChlD, MeChlM and MePrxQ under different concentrations of MgCl_2_ treatment. **a** The empty vector pET28a was expressed in *E. coli* as a control. The five concentration gradients of Mg^2+^ (0 mM, 50 mM, 100 mM, 150 mM and 200 mM) were performed. Data indicate mean ± SD, asterisks indicate significantly different compared to the control (Kruskal–Wallis test, *P* < 0.05). **b** The growth curve of the recombinant proteins MeChlD, MeChlM and MePrxQ under magnesium ion treatment. The growth curve is obtained by determining the OD_600_ of the bacterial solution under conditions of 200 mM MgCl_2_. **c** Drop plate test of recombinant protein MeChlD, MeChlM and MePrxQ under magnesium ion stress conditions. The drop plate test is obtained by determining the OD_600_ of the bacterial solution under conditions of 200 mM MgCl_2_

Magnesium as an essential nutrient for plant growth and development, involved in the photosynthesis process. To investigate the roles of MeChlD in photosynthesis, ChlM and PrxQ were selected as positive controls [39, 40]. These corresponding DNA were cloned into prokaryotic expression vector pET28a in order to expression them as recombinant proteins in *E. coli* BL21 (DE3). The five concentration gradients of Mg^2+^ (0 mM, 50 mM, 100 mM, 150 mM and 200 mM) were designed. The results indicated that the expression of MeChlD, MeChlM and MePrxQ proteins were significantly increased under 200 mM MgCl_2_ treatment (Fig. 2a). The growth curve showed that recombinant strain pET28a-MeChlD, pET28a-MeChlM and pET28a-MePrxQ were better than control strain pET28a under 200 mM MgCl_2_ treatment (Fig. 2b), and drip plate assay were also performed the growth of recombinant strain was better than control strain (Fig. 2c). These results suggest that Mg^2+^ highly affects the growth of the recombinant protein MeChlD *in E. coli*. Therefore, we predicated MeChlD may be play an important role in photosynthesis.

### Biological function of MeChlD in photosynthesis

**Fig. 3:**
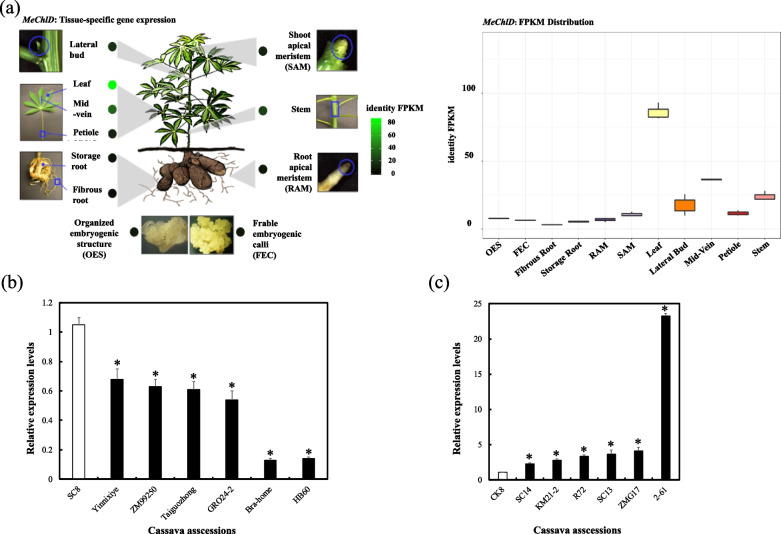
The profile of *MeChlD* expression in cassava. **a** Tissue-specific expression of *MeChlD* was analyzed in cassava gene expression atlas (http://shiny.danforthcenter.org/cassava_atlas/; Wilson et al., 2017). **b** *MeChlD* transcript levels in low photosynthetic rate of cassava accessions. **c** *MeChlD* transcript levels in high photosynthetic rate of cassava accessions. Leaves from cassava accessions were used for each RNA extraction (3 RNA extractions per variety; *n* = 3). Data indicate means ± SE. Statistically different transcript levels of different cassava accession compared with SC8 are marked by asterisks (the student’s test, *P* ≤ 0.05)

What is the biological role of *MeChlD* during the photosynthesis? To answer this question, the expression level of *MeChlD* was firstly analyzed. In this study, we found that *MeChlD* was expressed in the leaves, mid-veins and stems in cassava gene expression atlas, especially highly expressed in the leaves (Fig. 3a). And then the correlation between the expression level of *MeChlD* and the net photosynthetic rate in different cassava accessions was also characterized. In this study, 13 cassava accessions were selected, and classified into two categories according to the value of net photosynthetic rate (Table 1). The expression levels of *MeChlD* were strongly increased in high net photosynthetic rate of cassava accessions (Fig. 3b), on the contrary, *MeChlD* with low expression profiles in low net photosynthetic rate of cassava accessions (Fig. 3c). These results indicated that the expression profile of *MeChlD* have a significantly positive correlation with the net photosynthetic rate.

**Table 1:** Average net photosynthetic rate of 13 cassava accessions of national cassava germplasm repository (Danzhou) was detected

Cassava accessions	Accession numbers in NCGR database	Average net photosynthetic rate (μmol/(m^2^·s))	Classification
ZM99250	MS000094	15.32977475	Cassava accessions with low average net photosynthetic rate
TaiguoZhong	MS000626	15.5249856	
GR024-2	MS000245	15.66157154	
Bra-home	MS000526	15.66793562	
Yinnixiye	MS000021	15.87004708	
HB60	MS000623	16.01457674	
SC8	MS000129	16.9241	Control Plant
ZMG17	MS000441	17.79893448	Cassava accessions with high average net photosynthetic rate
SC13	MS000421	17.7997385	
KM21-2	MS000133	17.90160996	
R72	MS000126	18.30468849	
SC14	MS000226	19.1704428	
2–61	GPMS1000L	19.70053815	

**Fig. 4:**
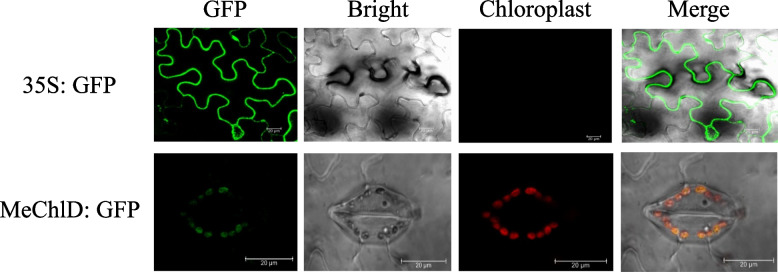
Subcellular localization of MeChlD in transiently transformed into the tobacco leaves. Bright field and fluorescence images were taken and merged. Scale bars represent 20 μm

To examined MeChlD localization, expressing MeChlD with C-terminal GFP tag was constructed. The fusion protein was transiently expressed in *N. benthamiana* leaves. Microscopy analysis indicated that MeChlD:GFP protein accumulated in the chloroplast after 3 dpi (Fig. 4). The result indicated that MeChlD was a chloroplast-localized protein.

### MeChlD physically interacts with MeChlM/PrxQ

ChlM and PrxQ play important functions in chlorophyll biosynthesis pathway and oxidative defense processes in chloroplasts, respectively. To analyze the interaction, the yeast two-hybrid system has been used. The vectors pGBKT7-*MeChlD*/pADT7, pGBKT7-*MeChlM*/pADT7 and pGBKT7-*MePrxQ*/pADT7 were constructed and transformed into yeast competent cell AH019. These combinations were able to grow in SD/-Trp-Leu dropout medium, but could not grow in SD/-Trp-Leu-Ade or SD/-Trp-Leu-Ade-His dropout medium. These results indicated that these vectors were expressed in yeast without auto-inducing (Figure S3).

**Fig. 5:**
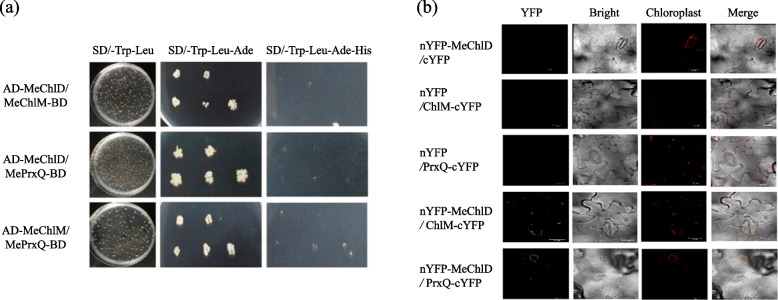
MeChlD interacts MeChlM and MePrxQ, respectively. **a** Analysis of the interaction between MeChlD-MeChlM and MeChlD-MePrxQ by the yeast two-hybrid assay. AD represents the pADT7-*MeChlM* and pADT7-*MePrxQ*; BD represents pGBKT7-*MeChlD* and pGBKT7-*MeChlM*. Interactions were assessed by growth on synthetic defined (SD) dropout medium lacking leucine and tryptophan (Leu/Trp), SD dropout medium lacking leucine, tryptophan and adenine (Leu/Trp/ Ade) and SD dropout medium lacking leucine, tryptophan, histidine and adenine (Leu/Trp/ Ade/His). **b** Bimolecular fluorescence complementation (BiFC) assays of the interaction between MeChlD-MeChlM and MeChlD-MePrxQ in tobacco leaves. Bright field and fluorescence images were taken and merged. Scale bars: 20 μm

The vectors pGBKT7-*MeChlD*/pADT7-*MeChlM*, pGBKT7-*MeChlD*/pADT7-*MePrxQ* and pGBKT7-*MeChlM*/pADT7-*MePrxQ* were constructed and transformed into yeast competent cell AH019. Specific interactions among MeChlD-MeChlM, MeChlD-MePrxQ, MeChlM-MePrxQ in yeast were confirmed (Fig. 5a), which were able to grow in SD/-Trp-Leu-Ade dropout medium. Remarkably, these interactions could not analyze in SD/-Trp-Leu-Ade-His dropout medium.

These vectors pGBKT7-*MeChlM*/pADT7-*MeChlD*, pGBKT7- *MePrxQ*/pADT7-*MeChlD* and pGBKT7-*MePrxQ*/pADT7-*MeChlM* were also constructed and transformed into yeast. The same results were observed (Figure S4). Therefore, we predicted that MeChlD-MeChlM, MeChlD-MePrxQ, MeChlM-MePrxQ showed weak interaction, respectively.

To further validate the interaction between MeChlD-MeChlM and MeChlD-MePrxQ observed in the Y2H system, the interaction of these proteins was tested using BiFC. The vectors pNC-BiFC-Enn-*MeChlD*, pNC-BiFC-Ecc-*MeChlM* and pNC-BiFC-Ecc-*MePrxQ* were constructed, and transformed into *A*. *tumefaciens* GV3101.

These fusion proteins were transiently expressed in tobacco leaves for 2–3 days. Microscopy analysis indicated co-expression of nYFP-MeChlD and MeChlM-cYFP, nYFP-MeChlD and MePrxQ-cYFP was observed yellow fluorescence signals at 3 dpi (Fig. 5b), when only expressed nYFP-MeChlD, MeChlM-cYFP, MePrxQ-cYFP was not observed any fluorescence signals, respectively. This observation was consistent with Y2H results. In conclusion, these results indicated that MeChlD can interact with MeChlM and MePrxQ, respectively.

### Silence of MeChlD by VIGS affecting chlorophyll synthesis, photosynthesis and starch accumulation

To find out the role of *MeChlD* during the photosynthesis in cassava, virus-induced gene silencing (VIGS) technology was used. Cassava variety SC9 was inoculated with *A. tumefaciens* carrying pCsCMV-*MeChlD*. After three wpi, the phenotype will be observed and taken a photo.

**Fig. 6:**
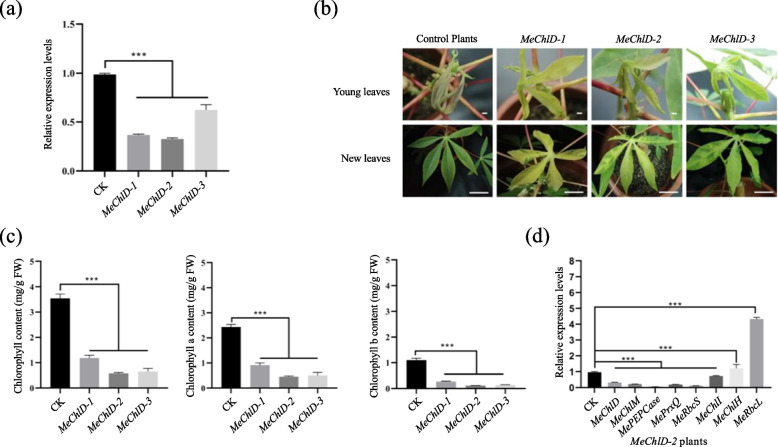
Comparison of phenotype between VIGS-*MeChlD* plants and control plants. **a** qRT-PCR analysis of the expression of *MeChlD* in the yellow leaves of VIGS-*MeChlD* plants (*MeChlD-1*, *MeChlD-2* and *MeChlD-3*). Leaves from VIGS-*MeChlD* plants were used for each RNA extraction (3 RNA extractions per silencing plants; *n* = 3). Data indicate means ± SE. Asterisks indicate significantly reduced compared to the control plants (the student’s test, *P* ≤ 0.05). **b** The VIGS-induce phenotype in leaves of VIGS-*MeChlD* plants in cassava at 3 wpi. Bars: 5 cm. **c** The content of total chlorophyll, chlorophyll a and chlorophyll b were measured in VIGS-*MeChlD* plants. Data indicate means ± SE. Asterisks indicate significantly reduced compared to the control plants (Kruskal–Wallis test, *P*  < 0.05). **d** qRT-PCR analysis of the transcript level of photosynthesis-associated nuclear genes in the yellow leaves of *MeChlD-2* plants. Data indicate means ± SE. Asterisks indicate significantly different compared to the control plants (the student’s test, *P* ≤ 0.05)

*MeChlD* expression was firstly analyzed by qRT-PCR in the leaves of VIGS-*MeChlD* plants at 3 wpi. These VIGS-*MeChlD* plants (*MeChlD-1*, *MeChlD-2* and *MeChlD-3*) showed significantly reduced *MeChlD* transcript levels (from 30–60%) in comparison with plants inoculated with empty vector pCsCMV-NC in cassava (Fig. 6a). The upper leaves without inoculated from VIGS-*MeChlD* plants altered from green to yellow color. The VIGS-induce phenotype of *MeChlD-1*, *MeChlD-2* and *MeChlD-3* exhibited fully yellow leaves, yellow sectors from mosaic leaves and green sectors from mosaic leaves at 3 wpi (Fig. 6b). However, the appearance in the yellow phenotype increased with time and spread over the entire leaf.

To characterize these silenced plants, chlorophyll content was measured. The content of chlorophyll a, chlorophyll b and total chlorophyll of *MeChlD-1*, *MeChlD-2* and *MeChlD-3* were significantly reduced compared to plants inoculated with empty vector pCsCMV-NC (Fig. 6c).

To explore the role of MeChlD in retrograde signaling, the photosynthesis-associated nuclear genes (PhANGs) were also analyzed in these VIGS-silenced plants. The expression genes involved in photosynthesis (*Rubisco small subunit*, *MeRbcS* and *phosphoenolpyruvate carboxylase*, *MePEPCase*) and the tetrapyrrole biosynthesis pathway (*ChlI* and *ChlM*) were significantly down-regulated of *MeChlD-2* plants (Fig. 6d), expect for *MeChlH* and *MeRbcL*. The transcript levels of PhANGs in the yellow leaves of *MeChlD-1* and *MeChlD-3* plants were also performed the similar results with *MeChlD-2* plants (Figure S5)*.* In conclusion, virus-induced gene silencing of *MeChlD* affects chlorophyll synthesis and photosynthesis in cassava.

**Fig. 7:**
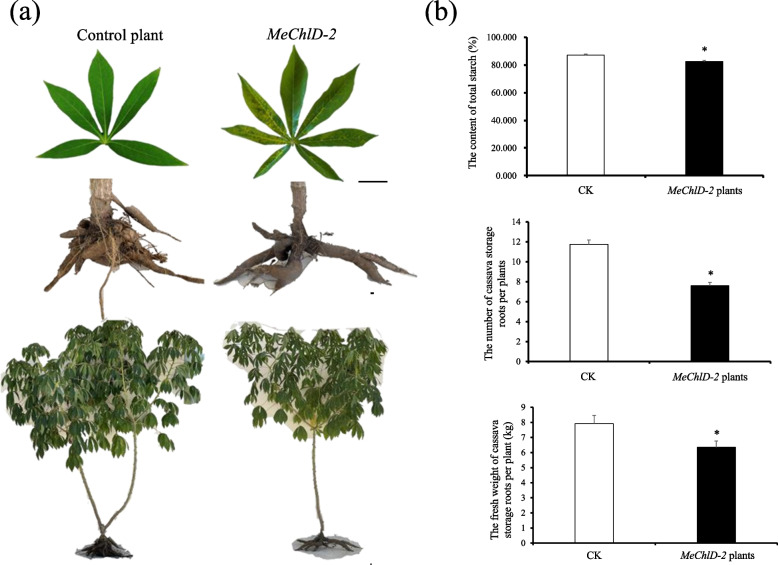
VIGS-*MeChlD-2* plants were transferred into the field and characterized after 4 months harvested. **a** The VIGS-induce phenotype in cassava leaves of *MeChlD*-2 plants in the field after 4 months harvested. Bars: 2 cm. **b** The storage root number, fresh weight and the content of total starch in cassava storage roots of *MeChlD-2* plants in the field were measured after 4 months harvested. Data indicate means ± SE. Asterisks indicate significantly reduced compared to the control plants (Kruskal–Wallis test, *P* < 0.05)

To further investigate the biological role of MeChlD during the source-to-sink process, VIGS-*MeChlD* (*MeChlD-2*) plants were further used and transferred into the field, and then harvested after 4 months. The phenotype of yellow leaves or mosaic leaves was also observed and maintained in *MeChlD-2* plants after 4 months in the field (Fig. 7a). The number and fresh weight of cassavas storage roots were significantly reduced in *MeChlD-2* plants compared to the control plants (Fig. 7b). Furthermore, the content of total starch in cassava storage roots of these VIGS-*MeChlD* plants was also significantly reduced in comparison to VIGS-NC control plants (Fig. 7b). These findings indicate that MeChlD involved in source-to-sink by affecting starch accumulation.

## Discussion

Photosynthesis directly affects crop production and quality [41]. Chlorophyll content is essential for photosynthesis [42]. Mg-chelatase is the first enzyme in the chlorophyll biosynthesis pathway. It catalyzes the insertion of magnesium ions into protoporphyrin IX under ATP-dependent conditions [17]. A subunit D is indispensable for the enzymatic reaction of Mg-chelatase in the activation and insertion steps [8]. However, to date, no *ChlD* has been characterized in Euphorbiaceae. In this study, a subunit D called *MeChlD* was successfully cloned and characterized in cassava cultivar SC9 (Figure S1). Alignment analysis and phylogenetic tree indicated that MeChlD protein belongs to AAA^+^ protein superfamily, and shows higher homology with HbChlD and RcChlD (Fig. 1). These results suggested that ChlD proteins are highly conservative in Euphorbiaceae. However, the biological function of ChlD is still unclear, thus requiring further elucidation in Euphorbiaceae, especially in tuberous crop cassava.

*ChlD* encodes one of the key enzymes in chlorophyll biosynthesis. In this study, a higher concentration of Mg^2+^ affected the growth of the recombinant protein MeChlD (Fig. 2). *MeChlD* was found to be highly expressed in leaves (Fig. 3a and b). Furthermore, the MeChlD protein is localized in the chloroplast (Fig. 4). Similar results were observed in other Mg-chelatase subunits of different plant species [17, 19, 40], suggesting that these subunits are involved in chlorophyll biosynthesis. In addition, Y2H assay indicated that MeChlD interacts with MeChlM and MePrxQ (Fig. 5a). However, the relationship of these interactions was not very strong (Fig. 5a). Therefore, further study was performed to elucidate the interactions (Fig. 5b). The results showed that MeChlD interacts with MeChlM to mediate Mg^2+^ transport, which is involved in chlorophyll biosynthesis. Meanwhile, MeChlD interacts with MePrxQ, which is involved in the oxidative defense process in chloroplasts. These results suggested that MeChlD not only plays an essential role in chlorophyll biosynthesis, but also involves with oxidative defense process in cassava.

Gene function analysis depends on the perfect genome. Genome sequencing of cassava with good quality has been completed and recently released [43]. However, most studies on cassava paid increased attention to omics research, without validating the gene function in depth, which is limited by the genetic transformation system [44]. Therefore, developing a powerful method for rapid analysis of gene function in vivo is necessary. VIGS system based on *cassava common mosaic virus* is widely used to investigate gene function in cassava [30, 32]. In this study, VIGS-induced reduction of *MeChlD* expression resulted in mosaic leaves or fully yellow leaves of VIGS-*MeChlD* plants compared with VIGS-NC control plants (Fig. 6b). This finding could be attributed to the reduction in chlorophyll content (Fig. 6c). These changes were consistent with those in previous studies on VIGS in Mg-chelatase subunits [17, 19, 30]. Furthermore, the changes in chlorophyll biosynthesis were significantly correlated with the reduced expression of photosynthesis-related genes (Fig. 6d), but not of *MeChlH* silencing. In this study, the expression of *MeChlI* was significantly reduced in VIGS-*MeChlD* plants, consistent with the result of previous studies, which showed that ChlI stabilizes ChlD, and acts as a chaperone [45]. The transcript level of *MeChlH* was significantly up-regulated in VIGS-*MeChlD* plants, and similar results have been reported in Arabidopsis *ChlM* mutant [46]. However, the different results indicated that ChlH accumulation without changing the expression of VIGS-*ChlD* plants in pea [17]. These findings suggested that the regulation of *ChlH* is a complex process.

To our knowledge, cassava is a typical source-to-sink tuberous crop, and its sink capacity depends on the delivery of forms of sources [47]. In this study, the phenotype of yellow leaves or mosaic leaves was observed after 4 months of plantation in the field (Fig. 7a). The content of total starch, the number and fresh weight of cassava storage roots (4 months) in VIGS-*MeChlD-2* plants significantly decreased compared with those in VIGS-NC control plants (Fig. 7b). This result indicated that the effect of VIGS could be maintained for at least 4 months in cassava storage roots, thus proving the hypothesis as described [30]. In conclusion, MeChlD not only possesses an important role in source biosynthesis, but also affects starch accumulation in cassava.

## Supplementary Information

**Additional file 1:** **Table S1.** Primers were used in this study.

**Additional file 2:** **Table S2.** The information of MV, pI and positive/negative charge residues of MeChlD. 

**Additional file 3:** **Figure S1.** *MeChlD* was successfully amplified in SC9. M: DL 15000 bp Marker; Line 1-3: the amplification bands of  *MeChlD CDS*

**Additional file 4:** **Figure S2.** Physical and chemical properties and structure analysis of protein. (a) MeChlD protein affinity and hydrophobicity prediction analysis chart. (b) Secondary structure of MeChlD protein. (c) Tertiary structure of MeChlD protein

**Additional file 5:** **Figure S3.** The auto-inducing activities of MeChlD, MeChlD and MerPrxQ were detected in yeast.

**Additional file 6:** **Figure S4.** The Y2H assay analysis of MeChlD,  MerPrxQ and MeChlD in vitro.

**Additional file 7:** **Figure S5.** qRT-PCR analysis of the expression of MeChlD, in the yellow leaves of *MeChlD-1* and  *MeChlD-3* plants.

## Data Availability

The sequence of MeChlD protein analyzed during the current study are available in the UniPort repository (Accession number: A0A2C9UIM7; https://www.uniprot.org/uniprotkb/A0A2C9UIM7/entry).

## References

[1] Cai J, Zhang J, Ding Y, Yu S, Lin HX, Yuan ZQ (2021). Different fertilizers applied alter fungal community structure in rhizospheric soil of cassava (*Manihot esculenta* Crantz) and increase crop yield. Front Microbiol.

[2] Zhu F (2015). Composition, structure, physicochemical properties, and modifications of cassava starch. Carbohyd Polym.

[3] De Souza AP, Massenburg LN, Jaiswal D, Cheng S, Shekar R, Long SP (2017). Rooting for cassava: insights into photosynthesis and associated physiology as a route to improve yield potential. New Phytol.

[4] Hailing Fu, Yi Qu, Pan Yi (2018). Efficiency of cassava production in China: empirical analysis of field surveys from six provinces. Appl Sci.

[5] Liang HB, Huang J, An FF, Wei YX (2016). Analysis of present situation of cassava industry in China. Acta Agriculturae Jiangxi.

[6] Alves AAC. “Cassava botany and physiology,” in Cassava: Biology, Production and Utilization, eds R. J. Hillocks, J. M. Thresh, and A. Bellotti (Wallingford: CABI), 2022:67–89.

[7] Yan W, Wu XY, Li YN, Liu GH, Cui ZF, Jiang TL (2019). Cell wall invertase 3 affects cassava productivity via regulating sugar allocation from source to sink. Front Plant Sci.

[8] Masuda T (2008). Recent overview of the Mg branch of the tetrapyrrole biosynthesis leading to chlorophylls. Photosynthesis Res.

[9] Zhou S, Sawicki A, Willows RD, Luo M (2012). C-terminal residues of *Oryza sativa* GUN4 are required for the activation of the ChlH subunit of magnesium chelatase in chlorophyll synthesis. FEBS Lett.

[10] Zhang H, Liu L, Cai M, Zhu S, Zhao J, Zheng T (2015). A point mutation of magnesium chelatase *OsCHLI* gene dampens the interaction between CHLI and CHLD subunits in rice. Plant Mol Biol Rep.

[11] Luo S, Luo T, Liu Y, Li Z, Fan S, Wu C (2018). N-terminus plus linker domain of Mg-chelatase D subunit is essential for Mg-chelatase activity in *Oryza sativa*. Biochem Bioph Res Co.

[12] Papenbrock J, Gräfe S, Kruse E, Hänel F, Grimm B (1997). Mg-chelatase of tobacco: identification of a *Chl* D cDNA sequence encoding a third subunit, analysis of the interaction of the three subunits with the yeast two-hybrid system, and reconstitution of the enzyme activity by co-expression of recombinant CHLD. CHLH and CHLI The Plant J.

[13] Papenbrock J, Pfündel E, Mock HP, Grimm B (2000). Decreased and increased expression of the subunit *CHLI* diminishes Mg chelatase activity and reduces chlorophyll synthesis in transgenic tobacco plants. The Plant J.

[14] Rissler HM, Collakova E, DellaPenna D, Whelan J, Pogson JB (2002). Chlorophyll biosynthesis. expression of a second *Chl I *gene of magnesium chelatase in *Arabidopsis* supports only limited chlorophyll synthesis. Plant Physiol.

[15] Kobayashi K, Mochizuki N, Yoshimura N, Motohashi K, Hisabori T, Masuda T (2008). Functional analysis of *Arabidopsis thaliana* isoforms of the Mg-chelatase CHLI subunit. Photoch Photobio Sci.

[16] Nakayama M, Masuda T, Bando T, Yamagata H, Ohta H, Takamiya KI (1998). Cloning and expression of the soybean *chlH* gene encoding a subunit of Mg-chelatase and localization of the Mg^2+^ concentration-dependent ChlH protein within the chloroplast. Plant Cell Physiol.

[17] Luo T, Luo S, Araújo WL, Schlicke H, Rothbart M, Yu J (2013). Virus-induced gene silencing of pea *CHLI* and *CHLD* affects tetrapyrrole biosynthesis, chloroplast development and the primary metabolic network. Plant Physiol Biochem.

[18] Zhang D, Chang E, Yu X, Chen Y, Yang Q, Cao Y (2018). Molecular characterization of Magnesium Chelatase in soybean  [*Glycine max* (L.) Merr.]. Front Plant Sci.

[19] Wu CJ, Wang J, Zhu J, Ren J, Yang YX, Luo T (2022). Molecular characterization of Mg-chelatase CHLI subunit in pea  (*Pisum sativum* L.). Front Plant Sci.

[20] Sawers RJ, Viney J, Farmer PR, Bussey RR, Olsefski G, Anufrikova K (2006). The maize *Oil yellow1* (*Oy1*) gene encodes the I subunit of magnesium chelatase. Plant Mol Bio.

[21] Mochizuki N, Brusslan JA, Larkin R, Nagatani A, Chory J (2001). *Arabidopsis genomes uncoupled 5* (*GUN5*) mutant reveals the involvement of Mg-chelatase H subunit in plastid-to-nucleus signal transduction. P Nat Acad Sci USA.

[22] Nott A, Jung HS, Koussevitzky S, Chory J (2006). Plastid-to-nucleus retrograde signaling. Annu Rev Plant Biol.

[23] Strand A, Asami T, Alonso J, Ecker JR, Chory J (2003). Chloroplast to nucleus communication triggered by accumulation of Mg-protoporphyrinIX. Nature.

[24] Tsuzuki T, Takahashi K, Inoue SI, Okigaki Y, Tomiyama M, Hossai MA (2011). Mg-chelatase H subunit affects ABA signaling in stomatal guard cells, but is not an ABA receptor in *Arabidopsis thaliana*. J Plant Res.

[25] Du SY, Zhang XF, Lu Z, Xin Q, Wu Z, Jiang T (2012). Roles of the different components of magnesium chelatase in abscisic acid signal transduction. Plant Mol Biol.

[26] Wang XY, LÜ  K, Cai  CP,  Xu  J, Guo  WZ (2014). Establishment and application of TRV-mediated virus-induced gene silencing in cotton. Acta Agronomica Sinica.

[27] Tian J, Pei HX, Zhang S, Chen JW, Chen W, Yang RY (2014). TRV-GFP: a modified tobacco rattle virus vector for efficient and visualizable analysis of gene function. J Exp Bot.

[28] Anu K, Jessymol KK, Chidambareswaren M, Gayathri GS, Manjula S (2015). Down-regulation of osmotin (*PR5*) gene by virus-induced gene silencing (VIGS) leads to susceptibility of resistant *Piper colubrinum* to the oomycete pathogen *Phytophthora capsici Leonian*. Indian J Exp Bot.

[29] Zeng H, Xie Y, Liu G, Wei Y, Hu W, Shi H (2019). *Agrobacterium*-mediated gene transient overexpression and *tobacco rattle virus* (TRV)-based gene silencing in cassava. Int J Mol Sci.

[30] Tuo D, Zhou P, Yan P, Cui H, Liu Y, Wang H (2021). A cassava common mosaic virus vector for virus-induced gene silencing in cassava. Plant Methods.

[31] Li X, Liu W, Li B, Liu G, Wei Y, He C, Shi H (2018). Identification and functional analysis of cassava DELLA proteins in plant disease resistance against cassava bacterial blight. Plant Physiol Biochem.

[32] An F, Xiao X, Chen T, Xue J, Luo X, Ou W, Li KM, Cai J, Chen SB (2022). Systematic analysis of bHLH transcription factors in cassava uncovers their roles in postharvest physiological deterioration and cyanogenic glycosides biosynthesis. Front Plant Sci.

[33] Artimo P, Jonnalagedda M, Arnold K, Baratin D, Csardi G, De Castro E (2012). ExPASy: SIB bioinformatics resource portal. Nucleic Acids Res.

[34] Wilson MC, Mutka AM, Hummel AW, Berry J, Chauhan RD, Vijayaraghavan A (2017). Gene expression atlas for the food security crop cassava. New Phytol.

[35] Kumar S, Stecher G, Li M, Knyaz C, Tamura K (2018). MEGA X: molecular evolutionary genetics analysis across computing platforms. Mol Biol Evol.

[36] Zhou SX. Kinetic and catalytic analysis of the magnesium chelatase in plants. doctoral dissertation, Wuhan: Huazhong agricultural university, China, 2012.

[37] Sparkes IA, Runions J, Kearns A, Hawes C (2006). Rapid, transient expression of fluorescent fusion proteins in tobacco plants and generation of stably transformed plants. Nat Protoc.

[38] Chaiareekitwat S, Latif S, Mahayothee B, Khuwijitjaru P, Nagle M, Amawan S, Müller J (2022). Protein composition, chlorophyll, carotenoids, and cyanide content of cassava leaves (*Manihot esculenta* Crantz) as influenced by cultivar, plant age, and leaf position. Food Chem.

[39] Xiao LG, Yang RC (2004). Molecular cloning and characterization of a stress-induced *peroxiredoxin Q* gene in halphyte *suaeda salsa*. Plant Sci.

[40] Wang Z, Hong X, Hu K, Wang Y, Wang X, Du S (2017). Impaired magnesium protoporphyrin IX methyltransferase (ChlM) impedes chlorophyll synthesis and plant growth in Rice. Front Plant Sci.

[41] Moss DN, Musgrave RB (1971). Photosynthesis and crop production. Adv Agron.

[42] Croce R, van Amerongen H (2014). Natural strategies for photosynthetic light harvesting. Nat Chem Biol.

[43] Hu W, Ji C, Shi H, Liang Z, Ding Z, Ye J (2021). Allele-defined genome reveals biallelic differentiation during cassava evolution. Mol Plant.

[44] Nyaboga E, Njiru J, Nguu E, Gruissem W, Vanderschuren H (2013). Unlocking the potential of tropical root crop biotechnology in east Africa by establishing a genetic transformation platform for local farmer-preferred cassava cultivars. Front Plant Sci.

[45] Lake V, Olsson U, Willows RD, Hansson M (2004). ATPase activity of magnesium chelatase subunit I is required to maintain subunit D in vivo. Eur J Biochem.

[46] Huang YS, Li HM (2009). Arabidopsis CHLI2 can substitute for CHLI1. Plant Physiol.

[47] Lemoine R, Camera SL, Atanassova R, Dédaldéchamp F, Allario T, Pourtau N (2013). Source-to-sink transport of sugar and regulation by environmental factors. Front Plant Sci.

