# Supplementary figures and images for: Magnesium chelatase subunit D is not only required for chlorophyll biosynthesis and photosynthesis, but also affecting starch accumulation in Manihot esculenta Crantz
Source: BMC Plant Biol. 2023 May 16;23:258. doi: 10.1186/s12870-023-04224-9 (PMC10186761; doi:10.1186/s12870-023-04224-9)

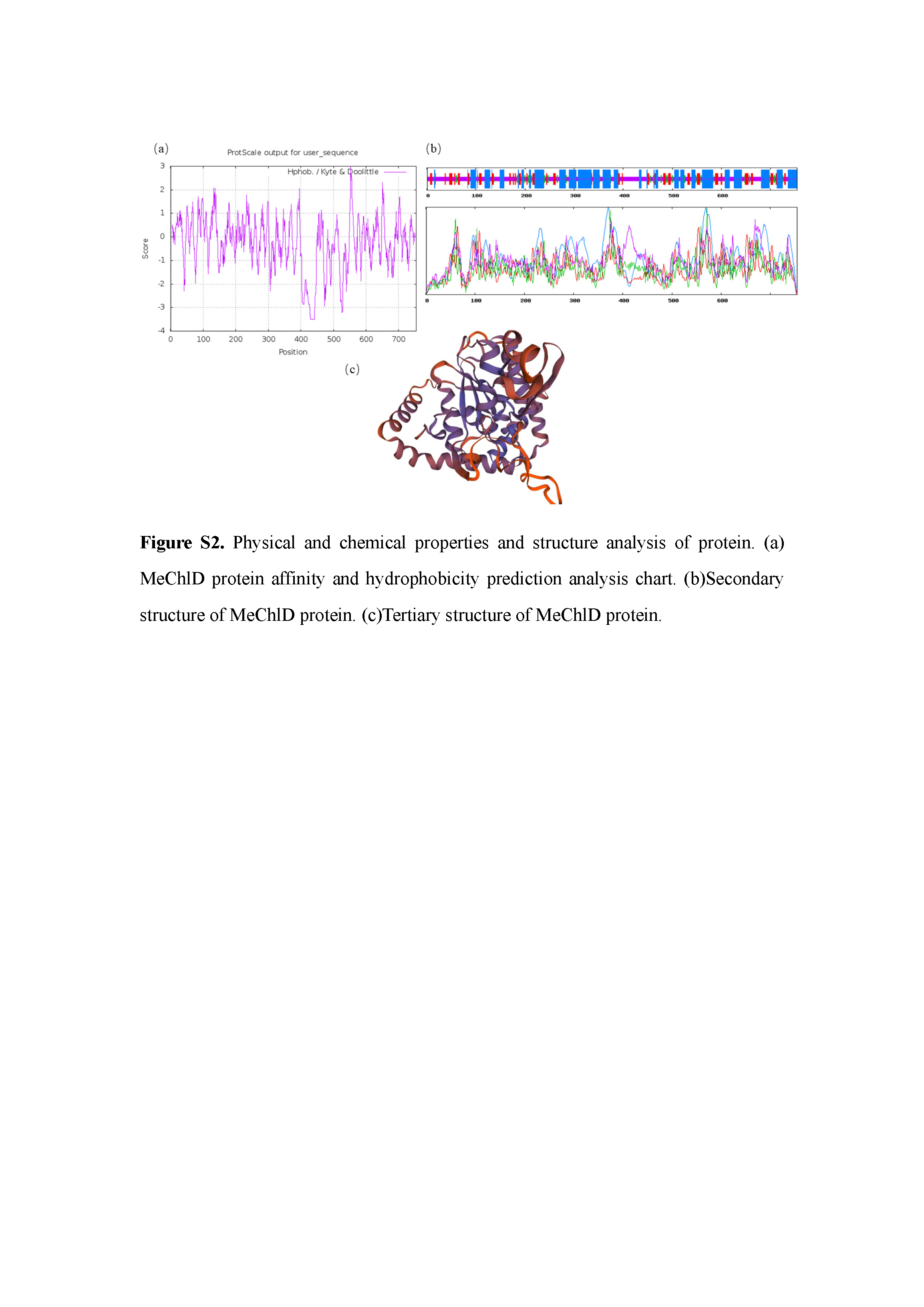

Supplement: Supplementary file 4 — Additional file 4: Figure S2. Physical and chemical properties and structure analysis of protein. (a) MeChlD protein affinity and hydrophobicity prediction analysis chart. (b) Secondary structure of MeChlD protein. (c) Tertiary structure of MeChlD protein [file 12870_2023_4224_MOESM4_ESM.tif]

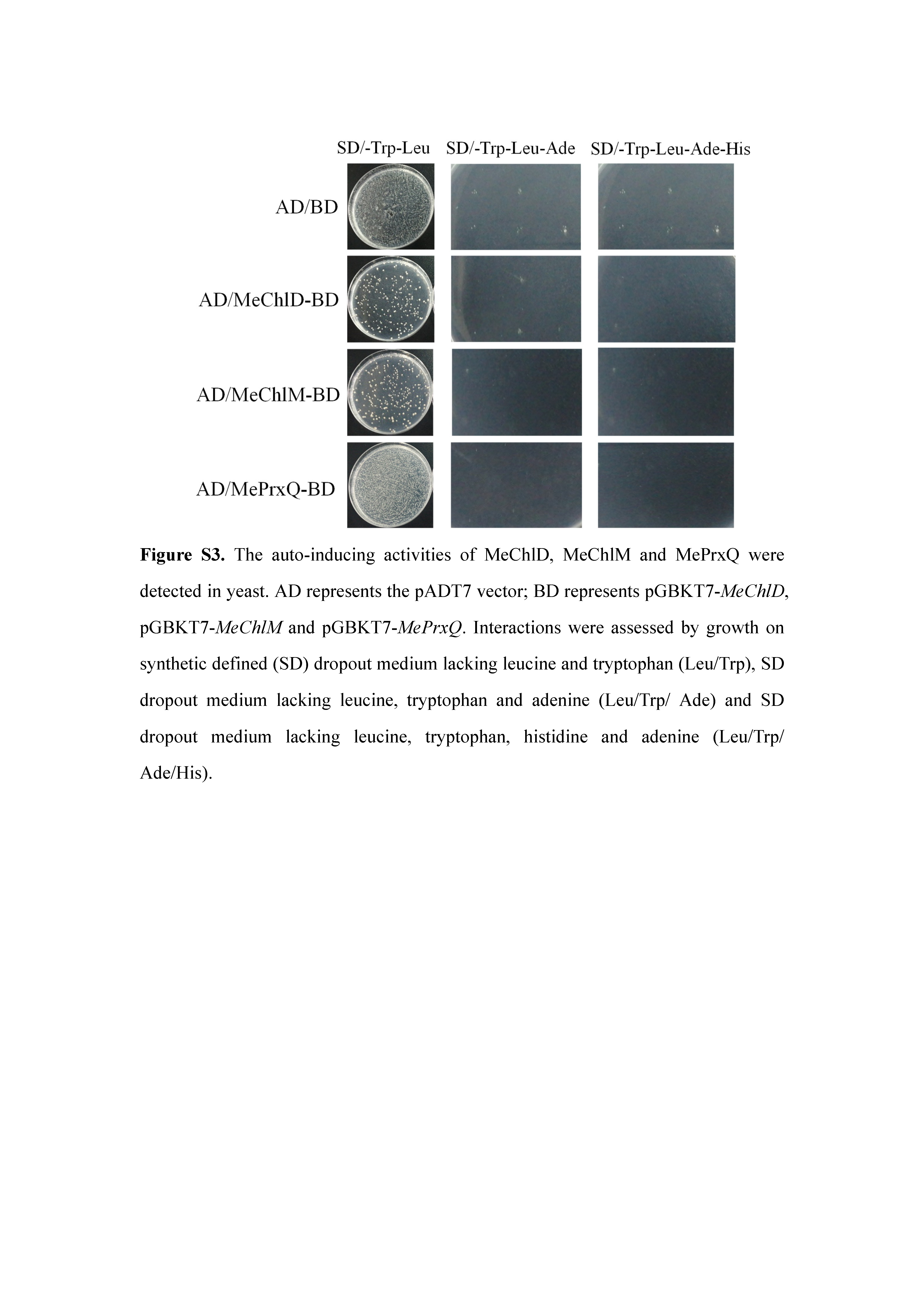

Supplement: Supplementary file 5 — Additional file 5: Figure S3. The auto-inducing activities of MeChlD, MeChlD and MerPrxQ were detected in yeast. [file 12870_2023_4224_MOESM5_ESM.tif]

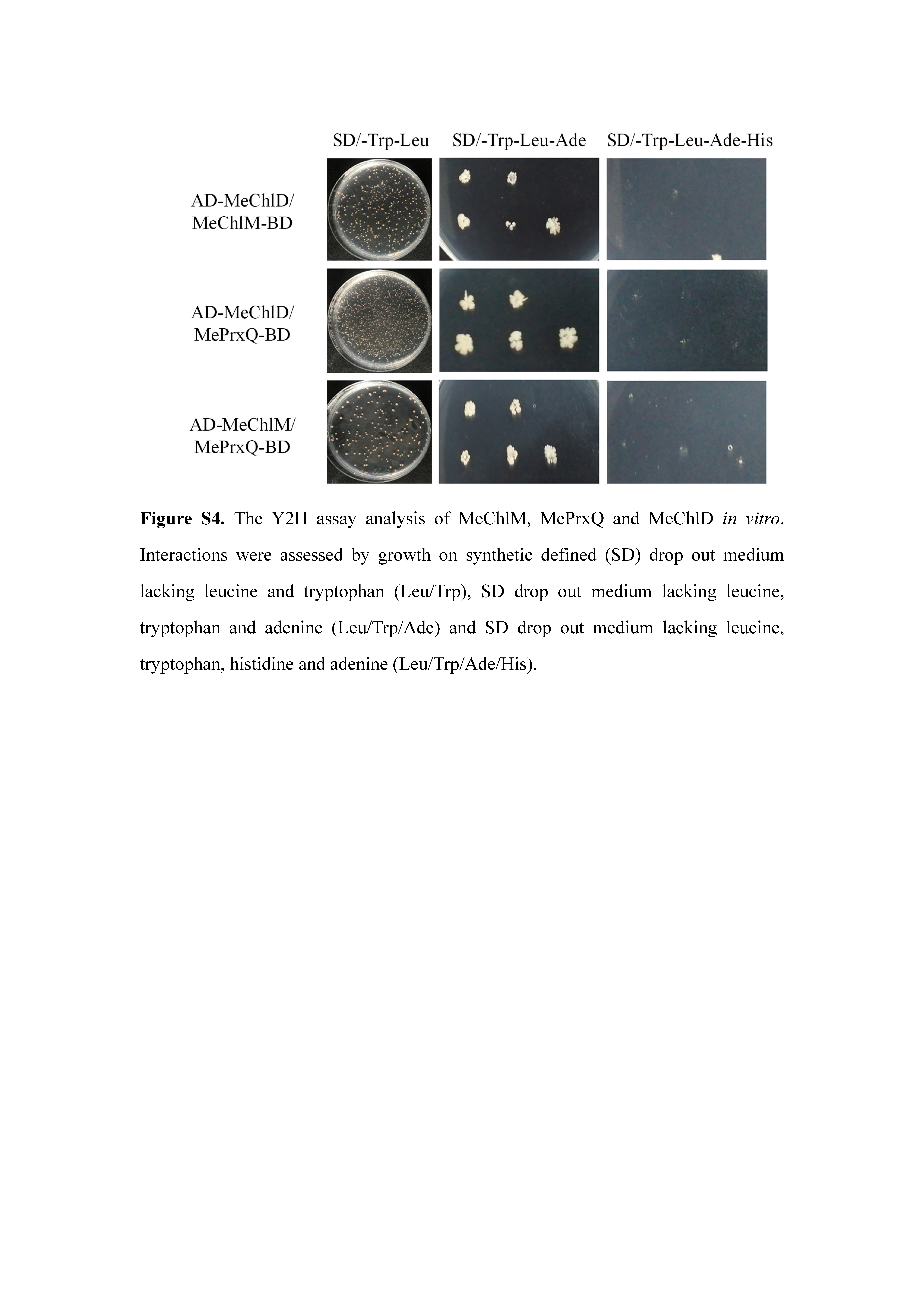

Supplement: Supplementary file 6 — Additional file 6: Figure S4. The Y2H assay analysis of MeChlD, MerPrxQ and MeChlD in vitro. [file 12870_2023_4224_MOESM6_ESM.tiff]

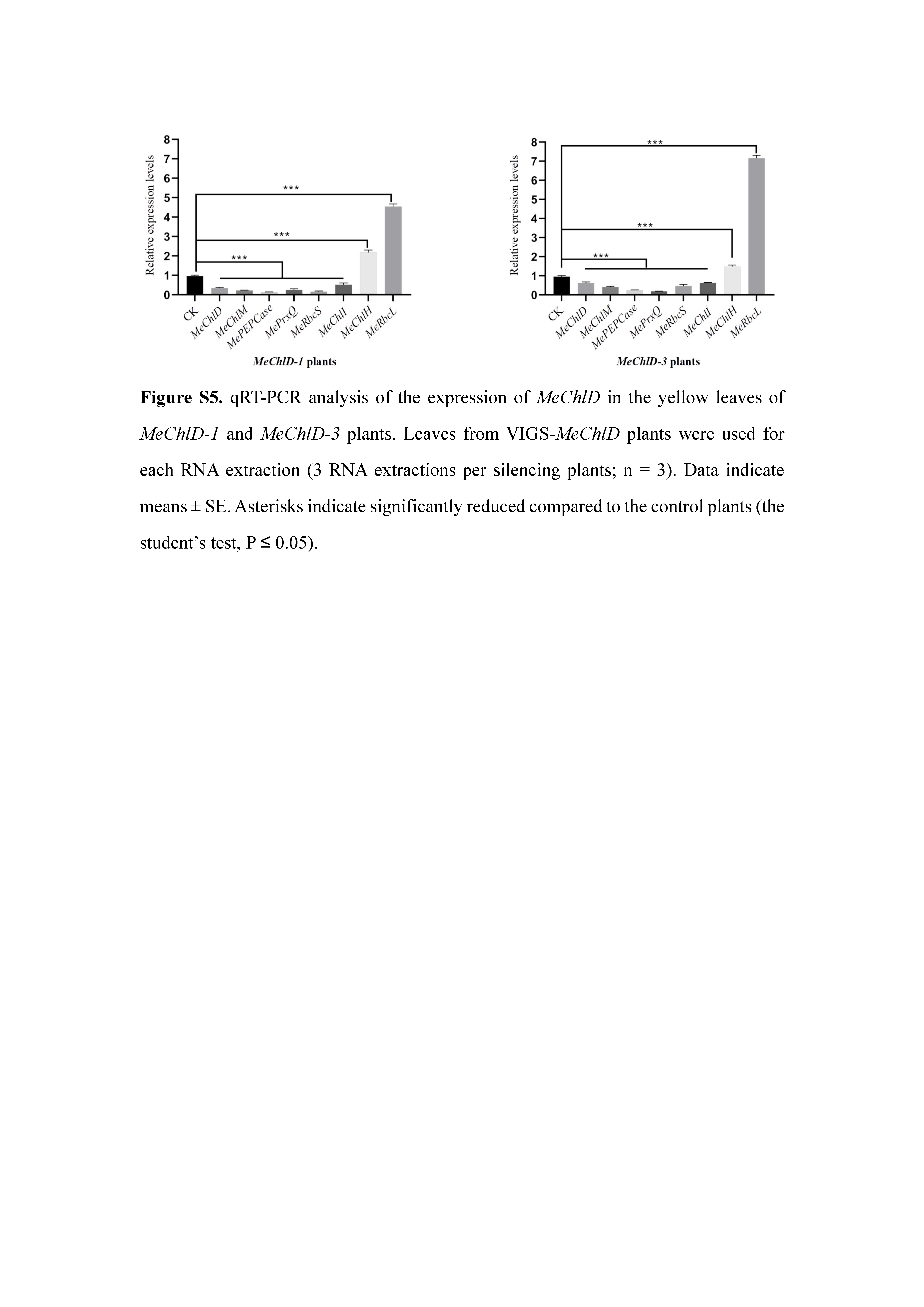

Supplement: Supplementary file 7 — Additional file 7: Figure S5. qRT-PCR analysis of the expression of MeChlD, in the yellow leaves of MeChlD-1 and MeChlD-3 plants. [file 12870_2023_4224_MOESM7_ESM.tif]
